# Prevalence and Management of Nonepithelial Ovarian Cancer in a Sub‐Saharan African Setting

**DOI:** 10.1155/ogi/2835994

**Published:** 2026-02-10

**Authors:** Biruck Gashawbeza Batu, Mekitie Wondafrash, Amanuel Yeneneh Teka, Don Eliseo Lucero-Prisno, Abraham Fessehaye Sium

**Affiliations:** ^1^ Department of Obstetrics and Gynecology, St. Paul’s Hospital Millennium Medical College, Addis Ababa, Ethiopia, sphmmc.edu.et; ^2^ Department of Research, St. Paul Institute for Reproductive Health and Rights, Addis Ababa, Ethiopia; ^3^ Department of Pathology, St. Paul’s Hospital Millennium Medical College, Addis Ababa, Ethiopia, sphmmc.edu.et; ^4^ Department of Management and Development Studies, University of the Philippines (Open University), Los Baños, Laguna, Philippines; ^5^ Department of Public Health and Policy, London School of Hygiene and Tropical Medicine, London, UK, lshtm.ac.uk

**Keywords:** germ cell tumors, nonepithelial ovarian cancer, sex cord–stromal tumors, yolk-sac tumors

## Abstract

**Background:**

Nonepithelial ovarian cancers (NEOCs) comprise a group of uncommon malignancies which can be challenging to treat. This broad term includes germ cell tumors, sex cord–stromal tumors, and rare types of ovarian cancer, such as small‐cell carcinomas and sarcomas. It is imperative that these rare tumors are managed with accurate diagnosis, staging, and treatment in order to optimize patient outcomes. The aim of this study was to describe the prevalence, pathology, and therapeutic interventions for NEOC in a Sub‐Saharan African setting.

**Methods:**

This is a 5‐year retrospective review of NEOC cases managed at St. Paul’s Hospital Millennium Medical College in Addis Ababa, Ethiopia, from September 2016 to September 2020. Data on NEOCs including clinical presentation, pathology, therapeutic interventions, staging status, type of surgery, histological subtype, and current disease status were extracted from patients’ records. Data were collected using a structured data extraction format. Data were analyzed using Stata release 15 (College Station, TX: StataCorp LLC).

**Results:**

The prevalence of NEOC was 17.3% (80 out of 264 cases of ovarian cancer). Among the types of NEOC, sex cord–stromal tumors were most common (46.2%) followed by germ cell tumors (43.8%). Of the germ cell tumors, yolk‐sac tumor was the common histologic subtype, representing 15% of all NEOC cases. Sixty‐five percent of cases were managed with staging surgery while 27% underwent fertility sparing surgery. There was no statistically significant association between patients’ age and type of tumor (*p* = 0.08).

**Conclusion:**

In this study, the prevalence of NEOC was 17.3%, which is higher than in other previous reports in the literature. Yolk‐sac tumor was the most common histologic subtype among germ cell tumors.

## 1. Introduction

Globally, ovarian cancer affects 239,000 patients and causes 152,000 deaths every year [1]. It remains the leading cause of death among gynecological cancers in most high‐income countries [2]. Proteomic technologies, such as mass spectrometry and protein array analysis, have greatly advanced the understanding of the molecular signaling pathways and the proteomic characterization of ovarian cancer. Proteomic analyses of ovarian cancer, as well as of tumor adaptive responses to therapy, can reveal new therapeutic targets that may help to reduce the development of drug resistance and ultimately improve patient outcomes [3]. There are still no effective tools for population‐wide screening with expensive overall care expenditure. Cost‐effective strategies for the early detection and prevention of ovarian cancer have been actively investigated over the past decade. Notably, the cost of treatment per patient with ovarian cancer remains highest among all cancer types [4]. Ovarian cancer is the third leading cause of cancer death among women in Ethiopia, with approximately 2550 diagnosed cases and 2000 deaths each year. The incidence and mortality rates of this disease have been increasing in Ethiopia and other parts of sub‐Saharan Africa over the past decade, likely due to changing lifestyle and reproductive factors [5].

Ovarian cancer encompasses a collection of neoplasms with distinct clinicopathological and molecular features and prognosis [6]. Type I epithelial ovarian cancers are suggested to be relatively indolent and genetically stable tumors that typically arise from recognizable precursor lesions, such as endometriosis or borderline tumors with low malignant potential. In contrast, Type II epithelial ovarian cancers are proposed to be biologically aggressive tumors from their outset, with a propensity for metastasis from small‐volume primary lesions. High‐grade serous cancers—the most common type of epithelial ovarian cancers, accounting for approximately 75% of epithelial ovarian cancers—develop according to the Type II pathway and present p53 and BRCA mutations [7]. Despite there being a variety of ovarian cancer subtypes, ovarian cancer cases in sub‐Saharan Africa are often treated as a single disease [6, 7]. NEOCs, which represent 10% of ovarian malignancies, can be differentiated in two major groups: germ cell tumors (GCTs) and sex cord–stromal tumors (SCSTs) [2, 5, 8]. GCTs differ to epithelial ovarian cancers with their earlier age of incidence, early‐stage disease (60%–70%), faster rate of growth, unilateral localization (95% of cases), and good prognosis [9]. Malignant GCTs represent 5% of all ovarian cancers and 80% of all preadolescent malignant ovarian tumors [10]. Currently, there is limited knowledge regarding ovarian GCT in postmenopausal patients. However, although rare, ovarian GCT should be considered in postmenopausal women presenting with an ovarian mass and elevated serum AFP levels [11].

SCSTs are rare neoplasms that account for approximately 3%–5% of ovarian malignancies and the majority of ovarian tumors with endocrine manifestations [12]. Unlike GCTs, SCSTs and steroid cell tumors are often unilateral and occur in patients across a wide age‐range. For example, granulosa cell tumors and thecomas are found mainly in peri‐ and postmenopausal women, whereas juvenile granulosa cell tumors, Sertoli cell tumors, and Sertoli–Leydig tumors usually develop in adolescents and in young females in whom fertility preservation is important [13, 14]. Yolk‐sac tumors (YSTs) structurally resemble the primitive yolk sac and display diverse histological patterns, including microcystic/reticular, endodermal sinus (festoon), solid, alveolar‐glandular, parietal, papillary, polyvesicular vitelline, hepatoid, and myxomatous types [15]. The aim of this study was to describe the prevalence, pathology, and therapeutic interventions for NEOCs at St. Paul’s Hospital Millennium Medical College (SPHMMC) in Addis Ababa, Ethiopia.

## 2. Methods

### 2.1. Study Setting and Design

This was a five‐year retrospective review conducted from September 2016 to September 2020 at SPHMMC in Ethiopia. SPHMMC is a national referral hospital in Ethiopia. It has various specialty and subspecialty care and training, including gynecologic oncology. In this study, we reviewed medical records for ovarian cancer cases who had primary NEOC.

### 2.2. Inclusion and Exclusion Criteria

The inclusion criteria were ovarian cancer cases who had a primary NEOC (germ cell or sex cord–stromal types), other rare metastatic tumors—such as MMT, Krukenberg tumors, and unspecified sarcomas—confirmed histology confirmation, and those with complete description of clinical characteristics including operation notes and pathology reports. We excluded those with incomplete data.

### 2.3. Data Collection

Data were collected from patient records and through phone call interview, using a structured questionnaire prepared in English. Data on NEOCs including clinical presentation, pathology, therapeutic interventions, staging status, type of surgery, histological subtype, and current disease status of the nonepithelial ovarian were extracted. Data were collected using a structured data extraction format.

### 2.4. Data Analysis

Data were analyzed using Stata release 15 (College Station, TX: StataCorp LLC). We employed simple descriptive statistics to assess frequencies, proportions, and *p* values. Bivariate analysis was used to determine the relationship between age and type of ovarian tumor.

### 2.5. Ethics Clearance

Ethical clearance was obtained from the Ethics Review Board of SPHMMC. The requirement to obtain informed consent from patients was waived by this ethics committee.

## 3. Results

The prevalence of NEOC among all ovarian cancer cases during the study period was 17.3% (*n* = 80). As demonstrated in Table 1, the majority patients were greater than 35 years old (27.5% were 36–50 years old, and 23.8% were greater than 50 years old). Among the types of NEOC, SCSTs were most common (46.2%) followed by GCTs (43.8%) (Table 2). Of the GCTs, YST was the most common histologic subtype, which represented 15% of the cases. Of the SCSTs, adult granulosa cell tumors were the most common, accounting for 91% of the cases.

**TABLE 1:** Age and parity of NEOC cases.

Characteristics (*n* = 80)	Frequency	Percentage (%)
*Age*		
Less than 15	6	7.5
16–25	17	21.3
26–35	16	20.0
36–49	22	27.5
> 50	19	23.8

*Parity*		
0	26	32.5
1–4	37	46.3
≥ 5	17	21.3

**TABLE 2:** Stage of cancer and distribution of histologic types.

Characteristics	Frequency	Percentage (%)
*Tumor stage (n = 80)*		
Stage I	38	47.50
Stage II	1	1.30
Stage III	16	20
Stage IV	3	3.80
Not staged	22	27.50

*Histologic types*		
*Germ cell tumors (n = 35)*	35	43.8
Yolk‐sac tumor	12	15.20
Dysgerminoma	8	10.10
Struma ovarii	5	6.30
Immature teratoma	4	5.10
Mixed germ cell tumors	3	3.80
SCC from mature teratoma	3	3.80

*Sex cord stromal (n = 37)*	37	46.2
Adult granulosa cell tumors	34	91.90
Poorly differentiated Sertoli Lyedig cell tumors	1	2.70
Mixed sex cord–stromal tumor with heterologous type	1	2.70
Fibro sarcoma	1	2.70

*Other rare types (n = 8)*	8	10
Krukenberg tumors	4	50
MMMT	2	25
Unspecified sarcomas	2	25

Sixty‐five percent of cases in this study were managed with staging surgery while 27% of them underwent fertility‐sparing surgery with limited staging (Table 3). In the remaining cases of advanced cancers, core needle biopsy and/or open biopsy was performed. Bowel resection and anastomosis were done in two cases that presented with bowel obstruction. As shown in Table 4, there was no statistically significant association between age and histologic type of tumor (*p* = 0.08).

**TABLE 3:** Type of surgery and current condition of the NEOC cases.

Surgery type	Frequency	Percentage (%)
Completely surgically staged	52	65
Salpingo‐oophorectomy	22	27.5
Biopsy taken	4	5
Others	2	2.5

*Current status*		
Alive	60	75
Alive with recurrence	2	2.5
Died	7	8.8
Unknown	11	13.8

**TABLE 4:** Age group distribution of nonepithelial ovarian cancers.

Age group	Type of tumor						*p* value
	Germ cell cancer		Sex cord stromal		Others		
	No.	%	No.	%	No.	%	
Less than 15	6.0	17.1	0.0	0.0	0.0	0.0	0.08
16–25	9.0	25.7	7.0	18.9	1.0	12.5	
26–35	7.0	20.0	8.0	21.6	1.0	12.5	
36–49	9.0	25.7	11.0	29.7	2.0	25.0	
> 50	4.0	11.4	11.0	29.7	4.0	50.0	

## 4. Discussion

In this study, more than one‐sixth of ovarian cancer cases were nonepithelial type. YST was the most common histologic subtype among GCT while adult granulosa cell tumors were the most common histologic subtype from the SCSTs. There was no association between patients’ age and histologic type.

According to recent literature, the prevalence of NEOC ranges from 8% to 10% [16, 17]. In our study, the prevalence of this cancer was 17.3%, which is higher than reports from previous studies. This could be explained by the reason that our center serves as a referral center for gynecology cases for other hospitals in Ethiopia, with the biggest number of experts in the field and who have high experience in managing such cases. Among the types of NEOC, SCSTs were the most common in our study (46% of all cases) followed by GCTs, which accounted for 43.8% of the total cases. This finding is contrary to reports from one earlier large study conducted in the Netherlands, which included 1258 NEOC cases and found 752 GCTs (60%), followed by 341 (27%) SCSTs and 165 (13%) sarcomas as the most common types [18]. Although it has been shown in most studies that dysgerminoma is the most common histologic subtype of GCT, in our study, YSTs were the most common, which accounted for 15% of all the GCTs [19]. This finding (high prevalence of yolk sac) warrants further future studies on this topic which should determine whether this is consistently true, as our study is the first to report on this topic from Ethiopia.

Fertility‐sparing surgery is the current standard of care for early‐stage NEOC. GCTs are typically unilateral, primarily affect young women and girls, and they are highly chemosensitive, with favorable prognosis [20, 21]. Importantly, second‐look surgery should be considered in patients with incompletely resected tumors containing teratomatous elements. In clinical practice, a second resection is generally reserved for patients with residual immature teratoma following adjuvant chemotherapy or in cases of growing teratoma syndrome [21].

In this study, 47% of cases had Stage I disease, and contrary to the recommendation for fertility‐sparing surgery, 65% of cases underwent staging surgery. The fact that 51.3% of these cases were for patients over 35 years old who might not have a desire for preserving fertility—we did not have cryopreservation services in our center during the study period—could be the possible explanation. In one recent study conducted in Sweden in 2019, 73 women aged 18–40 years with a Stage‐I NEOC diagnosis were investigated, and the majority of them (78%) underwent fertility‐sparing surgery, with only around 1 in 5 treated with radical staging surgery. The 5‐year overall survival rate, irrespective of surgical approach, was 98%. There were no statistically significant differences between overall survival and progression‐free survival rates in women treated with fertility‐sparing surgery, compared to radical surgery [15]. In our study, due to poor participants’ response and incomplete documentation, we did not perform survival analysis.

The main strength of this study is that it assesses an uncommon group of ovarian tumors in a unique low‐resource setting. This provides insight into the prevalence of these tumors as well as clinical practice patterns to treat them.

Limitations for this study include our inability to retrieve certain clinical characteristics of the study subjects due to the retrospective nature of the study. Small sample size and not applying proper sample size calculation precluded the possibility of doing advanced statistics such as regression analysis. Due to high incomplete response rate to our phone‐call interview in the years following treatment, we could not perform survival analysis with cruds ratio. This (not performing survival analysis with crude ratio with the available data) is one of the significant limitations of our study. Different histologic subtypes and unmatched staging of the cases also made it challenging to make clinically meaningful associations. Furthermore, important documentations such as peritoneal washings and lymph node assessment were missing in almost half of the GCT cases.

## 5. Conclusion

In this study, the prevalence of NEOC was 17.3%, which is higher than reported in previous literature. Unlike findings from previous studies, YSTs were the most common histologic subtype among GCTs. This finding should be further verified with future similar studies with larger sample size and multicenter design in Ethiopia and other sub‐Saharan Africa settings, as our study is among the first studies from sub‐Saharan Africa to present data on this topic—it documented the experience of treating NEOCs which could serve valuable lesson for other settings within the sub‐Saharan Africa.

## Author Contributions

Biruck Gashawbeza Batu contributed conception and development of the study project. Biruck Gashawbeza Batu and Amanuel Yeneneh Teka contributed data collection. Mekitie Wondafrash and Biruck Gashawbeza Batu performed the data analysis. Abraham Fessehaye Sium, Biruck Gashawbeza Batu, Don Eliseo Lucero‐Prisno III, Mekitie Wondafrash, and Amanuel Yeneneh Teka contributed manuscript write‐up. All authors accept responsibility for the paper as published.

## Funding

No funding was received for this study.

## Disclosure

All authors critically revised the article for intellectual content and gave final approval.

## Ethics Statement

Ethical clearance was obtained from the Ethics Review Board of St. Paul’s Hospital Millennium Medical College. The requirement to obtain informed consent from patients was waived by this ethics committee.

## Conflicts of Interest

The authors declare no conflicts of interest.

## Data Availability

Data are available from authors upon reasonable request.
